# Space-Time Patterns, Change, and Propagation of COVID-19 Risk Relative to the Intervention Scenarios in Bangladesh

**DOI:** 10.3390/ijerph17165911

**Published:** 2020-08-14

**Authors:** Arif Masrur, Manzhu Yu, Wei Luo, Ashraf Dewan

**Affiliations:** 1 The GeoVISTA Center, The Pennsylvania State University, University Park, PA 16802, USA; mqy5198@psu.edu; 2 10 Akron Street, Cambridge, MA 02138, USA; weiluo00001@gmail.com; 3 School of Earth and Planetary Sciences, Curtin University, Perth, WA 6102, Australia; a.dewan@curtin.edu.au

**Keywords:** SARS-CoV-2, COVID-19, coronavirus, infectious disease surveillance, emerging space-time pattern, disease tracking and monitoring, SaTScan

## Abstract

The novel coronavirus (COVID-19) pandemic continues to be a significant public health threat worldwide, particularly in densely populated countries such as Bangladesh with inadequate health care facilities. While early detection and isolation were identified as important non-pharmaceutical intervention (NPI) measures for containing the disease spread, this may not have been pragmatically implementable in developing countries due to social and economic reasons (i.e., poor education, less public awareness, massive unemployment). Hence, to elucidate COVID-19 transmission dynamics with respect to the NPI status—e.g., social distancing—this study conducted spatio-temporal analysis using the prospective scanning statistic at district and sub-district levels in Bangladesh and its capital, Dhaka city, respectively. Dhaka megacity has remained the highest-risk “active” cluster since early April. Lately, the central and south eastern regions in Bangladesh have been exhibiting a high risk of COVID-19 transmission. The detected space-time progression of COVID-19 infection suggests that Bangladesh has experienced a community-level transmission at the early phase (i.e., March, 2020), primarily introduced by Bangladeshi citizens returning from coronavirus epicenters in Europe and the Middle East. Potential linkages exist between the violation of NPIs and the emergence of new higher-risk clusters over the post-incubation periods around Bangladesh. Novel insights into the COVID-19 transmission dynamics derived in this study on Bangladesh provide important policy guidelines for early preparations and pragmatic NPI measures to effectively deal with infectious diseases in resource-scarce countries worldwide.

## 1. Introduction

The novel coronavirus (COVID-19) infection, caused by severe acute respiratory syndrome coronavirus 2 (SARS-CoV-2) [1], first emerged in December of 2019 in Wuhan city, China [2,3]. As of 11 August, 2020, there are more than 20 million confirmed cases and more than 735,000 deaths worldwide [4]. Countries around the world have been working to “flatten the curve” of this pandemic and many have seen a downward trend in the number of daily new cases, such as Italy and Spain. However, at the time of this study (i.e., mid-June, 2020), the COVID-19 pandemic was still strongly present with a daily upward curve in other countries, including Bangladesh. By mid-August, Bangladesh documented more than 264,000 confirmed cases in the country, with more than 152,000 recoveries and more than 3400 deaths. The daily reports by the Institute of Epidemiology, Disease Control and Research (IEDCR) suggests that Bangladesh detected its first three COVID-19 cases on March 8, 2020, when the number of confirmed cases already surpassed 100,000 worldwide (WHO statement, 2020). While substantial knowledge has been accumulating on the transmission dynamics of this pandemic in countries with early-state outbreak (e.g., China, Italy, United States) [5,6,7], relatively little is currently known of the spatio-temporal outbreak dynamics of this pandemic in Bangladesh, where it is still unfolding.

**Figure 1:**
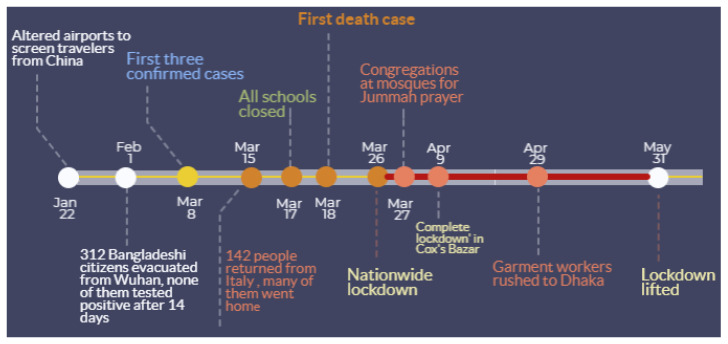
Major COVID-19-related events in Bangladesh and non-pharmaceutical intervention (NPI) measures by the government.

While public and private sectors in Bangladesh keep fighting to cope with the ramifications of this deadly pandemic, the scientific communities are contributing toward improving existing understanding on various aspects of this disease [8,9,10,11,12]. Among very few studies on COVID-19 spread in Bangladesh, Kalam and Hussain (2020) [13] used analytical models (e.g., logistic) for analyzing an early growth dynamics of disease transmission. Their findings suggest that early growth of COVID-19 cases followed an exponential pattern with an estimated 5.16 days doubling period. A recent study [14] reported that the COVID-19 trend in Bangladesh showed deviation from the epidemiological model, and they identified potential factors responsible for this deviation. Overall, Bangladesh’s COVID-19 characteristic patterns reflect the following: an inadequate testing and treatment capacity, insufficient supply of personal protection equipment, poor public sanitation practices, poorly managed public health care system and services, lack of public awareness, large-scale violation of social distancing order (Figure 1, Figure 3, Figure A2 and Figure A3), a large vulnerable population (e.g., Rohingya refugees), community-level transmission, and dire economic impacts [15]. While existing research and unpublished reports highlight these important aspects of the COVID-19 outbreak in Bangladesh, knowledge is largely missing on the geographic perspectives of the transmission dynamics and intervention scenarios in the country.

Spatial, temporal, and spatio-temporal statistics are essential approaches for identifying transmission dynamics of COVID-19, which can enable evaluating ongoing efforts as well as inform new, innovative solutions for containing the disease [16,17]. Existing literature shed light on the spatiotemporal dynamics of COVID-19, which helped identify the extent and impact of the pandemic as well as intervention strategies in different parts of the world including China [2,18,19], Italy [20], Spain [21], Brazil [22], and the United States [5,23,24]. In this paper, we leveraged a prospective space-time scan statistic approach [25] to detect currently “active” and emerging clusters of COVID-19 outbreak in Bangladesh. For that, we utilized spatio-temporally aggregated daily confirmed cases made available by the IEDCR. Additionally, we conducted tracking analysis using confirmed cases of a cumulative 1-week progression (i.e., to account for incubation period) for mapping the spatial propagation of the disease outbreak to understand COVID-19 transmission dynamics in both space and time. Finally, we identified potential time-lagged (i.e., characterizing incubation period) links between the space-time outbreak and NPI scenarios based on available governmental and newspaper reports.

Specifically, this study has following contributions to the understanding of COVID-19 transmission dynamics relative to NPI efforts in the example of Bangladesh:

1. Timely detection and evaluation of active space-time clusters of COVID-19 infections that currently pose threat to public health;
2. Track space-time propagation of previously existed clusters for an understanding of ‘where’ and ‘when’ infections spread;
3. Identify linkages between the retrospective emerging patterns of COVID-19 spread and time-lagged NPI scenarios.

## 2. Materials and Methods

### 2.1. COVID-19 Daily Reports—Bangladesh and Dhaka Megacity

**Figure 2:**
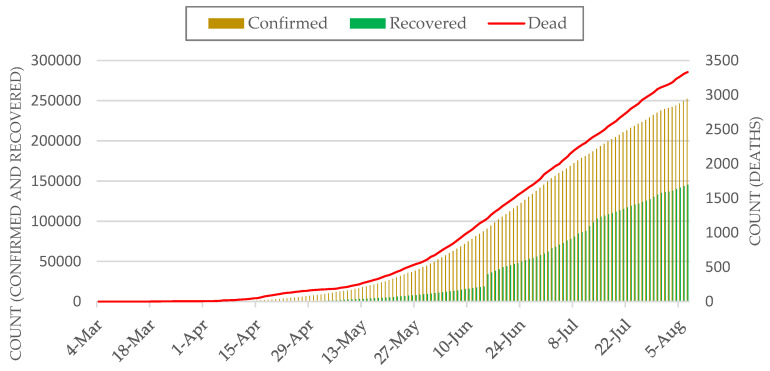
Cumulative count of COVID-19 cases in Bangladesh.

We used freely available COVID-19 daily reports from the IEDCR website (https://www.iedcr.gov.bd/) that contain cumulative counts of confirmed cases over the past 24 h nationwide. Note that, our study could not incorporate data on COVID-19 cases beyond 15 June because such data at the administrative levels (e.g., district) were not publicly available. At the time of this study (mid-June, 2020), daily confirmed cases were on the rise in Bangladesh, particularly in Dhaka megacity. Although district-level reported cases were made available since 4 March by the IEDCR, currently location-based (i.e., place of the patient’s resident) daily counts are only available from 4 April Bangladesh and 7 April for Dhaka. Figure 2 shows the most recent accounts of cumulative numbers of confirmed, recovered, and death cases at the country level.

**Figure 3:**
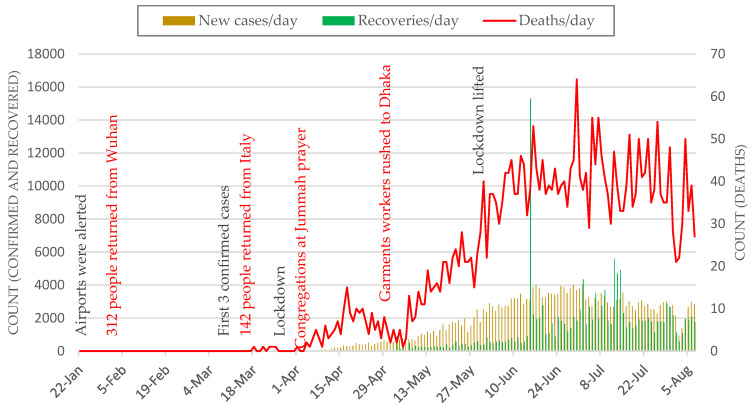
Daily new cases of COVID-19 cases in Bangladesh. Red labels show major events related to violations of NPI measures.

Our emerging cluster analyses required counts of new cases per temporal unit (e.g., day) at different geographic locations—i.e., districts and thanas. Therefore, we subtracted daily cumulative counts from the previous day’s count to obtain the present day’s new cases for the entire available timeline. Figure 3 shows the most recently available daily counts of newly confirmed, recovered, and death cases over the past 24 h at the country level (i.e., sum of all districts per day).

### 2.2. LandScan Population Data

Our analysis required the specification of population size over space in each spatial unit. For that, we used the LandScan™ Population dataset with a ~1 km spatial resolution [26,27]. The currently available 2018 LandScan dataset of Bangladesh was used to extract total population count at the thana level for Dhaka city. Note that, our district-level analysis used the population count that was inherently present with the COVID-19 dataset for Bangladesh provided by IEDCR’s GIS dashboard (IEDCR, 2020).

### 2.3. Emerging Space-Time Cluster Analysis

We detected currently emerging space-time clusters of COVID-19 cases in Bangladesh using the prospective space-time scan statistic [28,29]. For our analyses, we used SaTScan (version. 9.6) [30]. The SaTScan statistic has been regularly used in epidemiology to identify significant clusters of diseases. Using different models (e.g., Poisson, Bernoulli), it detects and maps unexpected space-time clustering given baseline conditions (e.g., population size). This statistic is defined by a cylinder-shaped window with a round spatial base and with height analogous to time. The base of the cylinder is centered on centroids of the geographic unit (e.g., district) throughout the study area (i.e., Bangladesh). The radius of the base varies continuously in size until a maximum upper bound is reached. The height denotes time interval of <= 50% of the study period, as well as the study period as a whole. The cylindrical window is moved in space and time to cover each possible geographic location and size, as well as each possible time interval.

The prospective space-time scan statistic detects active clusters of COVID-19 infections that are still being found at the end of the study period. Table 1 shows input parameters used in our prospective analyses. We used a discrete Poisson-based probability model on the assumption that the COVID-19 cases follow a Poisson distribution according to the at-risk population in Bangladesh. The model also assumes the population size to be static in an area over a time period. The null hypothesis H_0_ stipulates that the model represents a diverse Poisson process of contracting COVID-19 with an intensity μ (proportional to the at-risk population), whereas the alternative hypothesis H_A_ supports that the number of observed cases is greater than the number of expected cases. The following equation calculates an expected number of COVID-19 cases (μ) [5,31]:

(1) $$\mu =p*\;\frac{C}{P}$$

where p is the population in a geographic area (e.g., district or thana); C and p are the total COVID-19 cases and the total estimated population in Bangladesh, respectively.

For each cylinder, the number of infections inside and outside the cylinder, as well as the expected number of cases reflecting the population at risk and relevant covariates, are used to calculate the likelihood for each cylinder. The most likely cluster has a cylinder with the maximum likelihood and more than the expected number of cases. Equation (2) was used for calculating maximum likelihood ratio that identified scanning windows with elevated risk for COVID-19 [29].

(2) $$\frac{L\left(Z\right)}{L_{0}}=\frac{{\left(\frac{n_{Z}}{\mu \left(Z\right)}\right)}^{n_{Z}}{\left(\frac{N-n_{Z}}{N-\mu \left(Z\right)}\right)}^{N-n_{Z}}}{{\left(\frac{N}{\mu \left(T\right)}\right)}^{N}}$$

where L(Z) is the likelihood function for Cylinder Z and L_0_ is the likelihood function for H_0_; n_Z_ is the number of COVID-19 cases in a cylinder; μ(Z) is the number of expected cases in Cylinder Z; N is the total number of observed cases for the entire study areas—Bangladesh or Dhaka citys—across all time periods; and μ(T) is the total number of expected cases in the study area across all time periods. Risk elevates for the cylinder when it has a likelihood ratio greater than 1.

Multiple geographic units can belong to a significant space-time cluster, which assumes that the relative risk (RR) of COVID-19 is homogeneous across a cluster. To avoid that assumption, we derived relative risk for each geographic unit—district and thana—that lies within a cluster. The RR is calculated using Equation (3) [31,32]:

(3) $$\mathrm{RR}=\frac{c/e}{\left(C-c\right)/\left(C-e\right)}$$

where c is the total number of COVID-19 cases in a district, e is the total number of expected cases in a district, and C is the total number of observed cases in Bangladesh, for example. RR is thus an estimated risk within a location divided by the risk everywhere else. Clusters also have RR, which is calculated using same way as in Equation (3). For example, an RR value of 5.5 for a location or a cluster suggests that the population within this location or cluster is 5.5 times more likely to be exposed to COVID-19 infections compared outside of that location or cluster.

In the following section, we present active and emerging clusters of COVID-19 cases in the Dhaka city and all 64 districts of Bangladesh up to 15 June. To elucidate the space-time transmission dynamics of COVID-19 cases in Bangladesh relative to NPI scenarios, first, we divided current timeline of COVID-19 cases into two groups: 4 April to 30 April and 4 April to 15 June. 30 April was used as a cut-off date as it signifies one of the major breaks in social distancing over the past two weeks (Figure A2), which may have led to the emergence of new active clusters. Second, for an improved understanding of the space-time propagation of relative risk across districts in Bangladesh, we detected emerging clusters using a cumulative 1-week prospective scanning approach. This enabled considering a roughly 1 to 2-week incubation period for locating dissipating risk and newly emerged high-risk areas along the timeline. Finally, we analyzed the linkages between these clusters and known NIP scenarios in space and time.

## 3. Results

### 3.1. Thana-Level Emerging COVID-19 Risk Clusters in Dhaka Megacity

Being the capital and largest industrial and economical hub of the country, Dhaka experienced the inception of the COVID-19 outbreak in Bangladesh. Although the Bangladesh government-initiated intervention measures in mid-March (see Figure 1), it was not until 26 March when the entire nationwide lockdown was put in place and extended until 31 May 2020. Meanwhile, other intervention measures were neglected by the authorities and general public [33], which may have resulted in the transmission of COVID-19 across and beyond Dhaka city.

**Figure 4:**
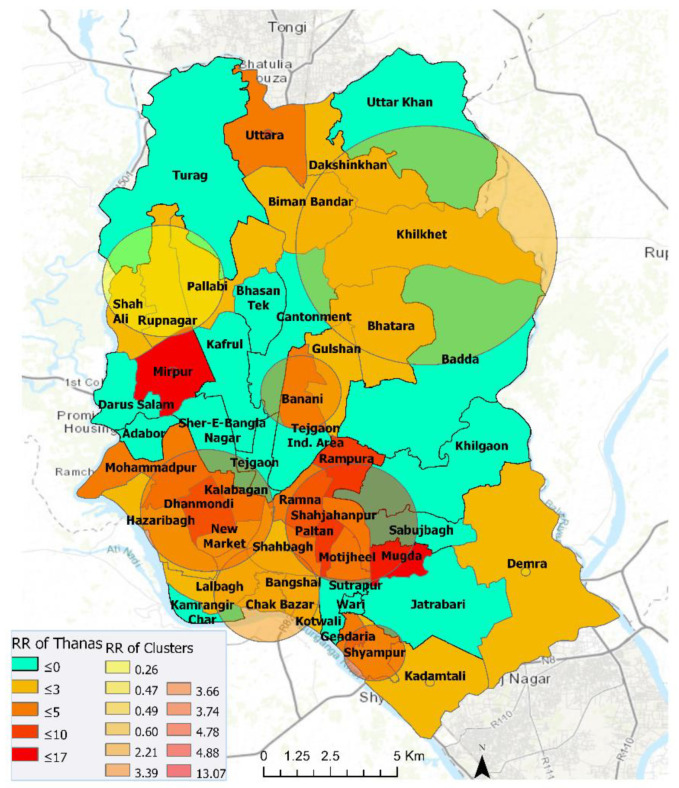
Emerging space-time clusters at the thana-level in Dhaka city (7 April–15 June 2020).

**Figure A1:**
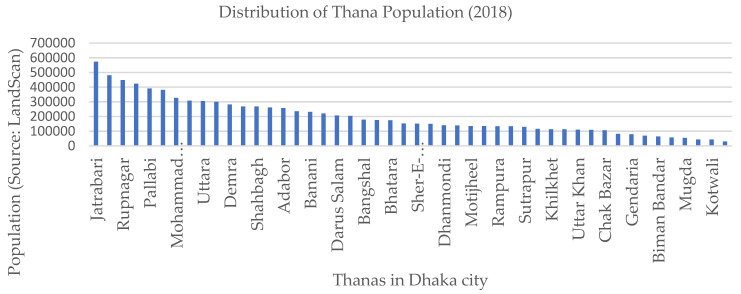
Distribution of the Thana population of 2018 in Dhaka megacity, Bangladesh.

**Figure A2:**
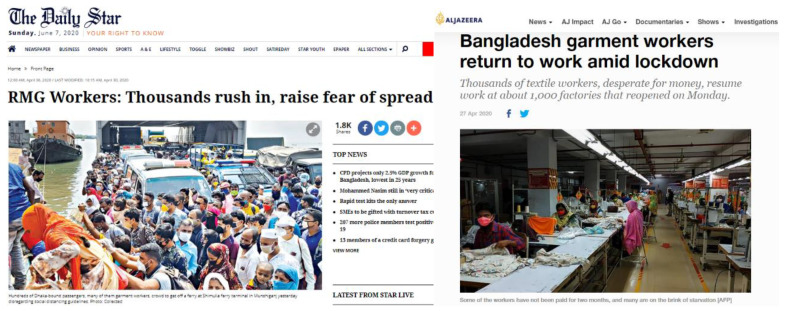
Snapshots of print media reports between 27 April and 30 April when a large number of people in Bangladesh congregated outside amidst nationwide lockdown.

We found eleven statistically significant emerging space-time clusters of COVID-19 at the thana level in Dhaka megacity from 7 April to 15 June, 2020. Table 2 shows that, among these clusters, there were twenty-three thanas with a higher relative risk (RR > 1), where there were more observed than expected coronavirus cases over the timeline based on the parameters set in Table 1. These high RR cluster areas vary in size and magnitude of risk. Lower RR (< 1) areas are characterized by higher expected than observed cases. The relationship between population density and relative risk suggests that all locations in Dhaka have not responded evenly to the outbreak. For example, the emerging cluster with the highest RR (11.94) is found in Mirpur with a relatively moderate population size (Figure 4 and Figure A1). Other areas within Pallabi and Rupnagar thana that are relatively more populated and lower to medium income were experiencing a relatively high level of RR (4.42). The northernmost (i.e., Uttara thana) areas in Dhaka city were exhibiting smaller radius of emerging hotspot but with higher magnitudes. The onset of these “active” clusters took place between early May and mid-June. However, clusters that emerged early (12 May–15 June) were exhibiting lower risk, whereas relatively younger clusters (30 May–15 June) demonstrated a higher risk of outbreak. At the same time, many areas were exhibiting low to no risk by mid-June, which were high-risk areas over the early April to mid-May period. A weekly or bi-weekly prospective scanning will reveal the space-time propagation of COVID-19 hotspots in Dhaka city. Since our primary focus is on elucidating district-level transmission dynamics, in the next section we have demonstrated space-time emerging patterns and propagation of COVID-19 for the entire country.

### 3.2. District-Level Emerging COVID-19 Risk Clusters—4 April–15 June 2020

As mentioned previously, travel to and from Dhaka city, before and during the nationwide lockdown, may have propagated a community-level transmission in other parts of the country. Hence, in the next sections, we show results from district-level analyses for two distinct periods that characterize large-scale NPI violations and increase in reported COVID-19 cases.

#### 3.2.1. 4 April–30 April 2020

**Figure 5:**
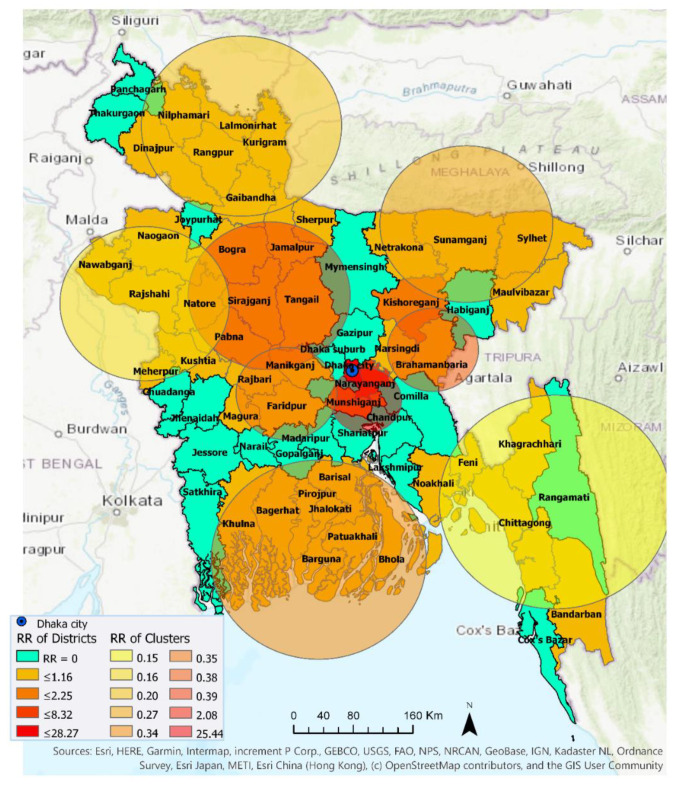
Emerging space-time clusters at the district level between 4 April and 30 April 2020.

**Figure 6:**
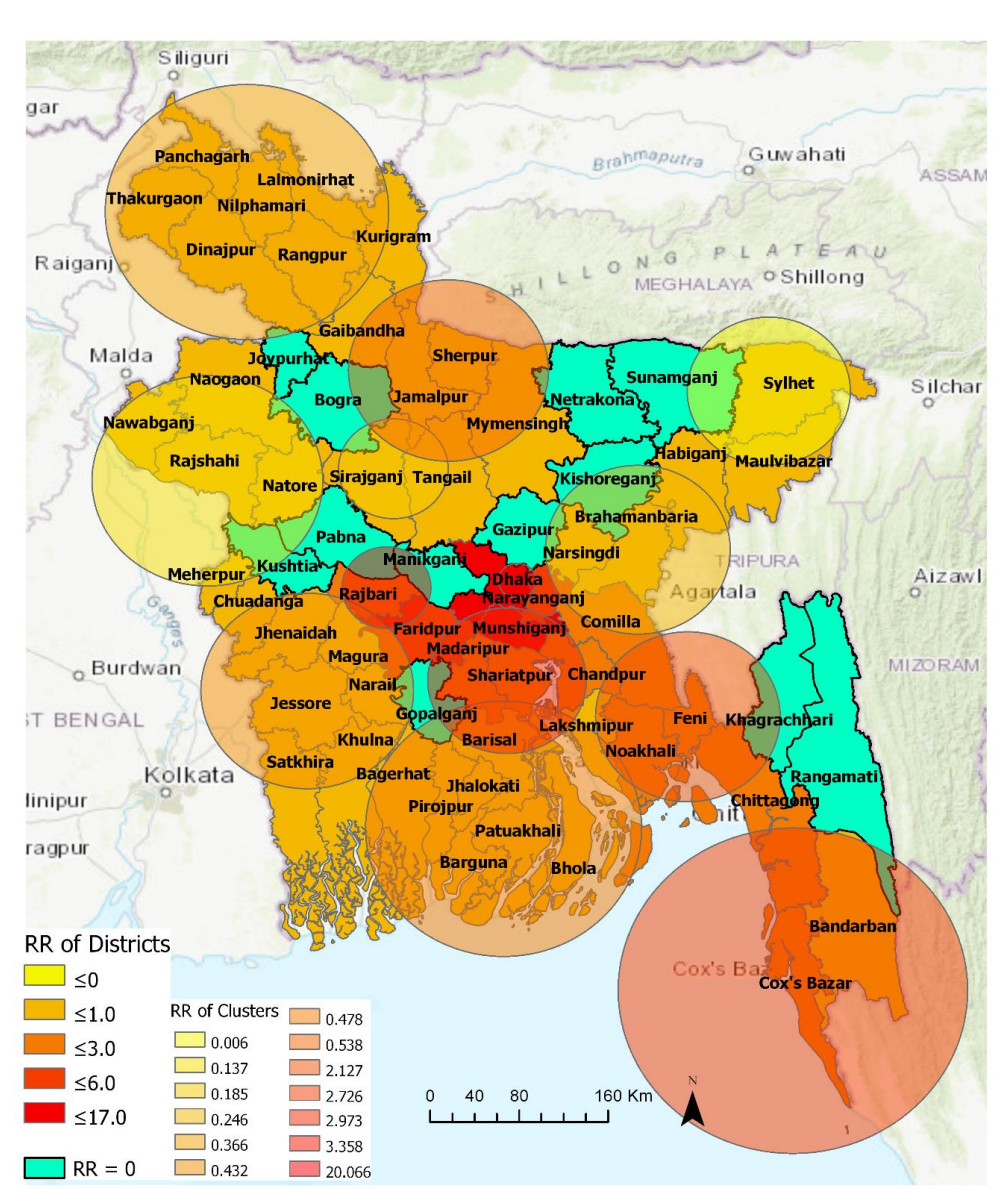
Emerging and non-emerging space-time clusters at the district level between 4 April and 15 June 2020.

Ten statistically significant emerging space-time clusters were detected in Bangladesh between 4 April and 30 April. Table 3 shows characteristics of these clusters. There were two high-risk clusters with RR > 1 (i.e., more observed cases than expected) and eight low-risk clusters with RR < 1 (i.e., more expected than observed) across a total of 46 districts with at least one confirmed COVID-19 case. During this period, 20 districts out of total 64 districts in Bangladesh exhibited no relative risk of exposure (RR = 0) to the COVID-19 infection (Figure 5). Cluster 1, containing Dhaka (metropolitan area), Narayanganj, and Munshiganj, had an RR of 25.44, which suggests that the population within this cluster was a record 25.44 times more likely to be exposed to COVID-19 compared to other clusters. Cluster 9 contains only one district, Kishoreganj, with an RR of 2.08. The other eight clusters had an RR less than 1, suggesting that populations within these cluster locations were exposed to a very low risk of coronavirus infection. Cluster 10, which included two districts—Narsingdi and Brahamanbaria—exhibited smaller and a relatively delayed time period (22 April–30 April) than other clusters. Note that, this low-risk exposure in Brahamanbaria elevated to a higher-risk exposure in the following week (Figure 6 and Figure 8), which altogether can be linked to a public event in the previous week (on 18 April) that violated the government-imposed social distancing order. Figure 5 visualizes locations of high and low RR districts within these ten clusters.

#### 3.2.2. 4 April–15 June 2020

Thirteen statistically significant emerging space-time clusters were detected in Bangladesh between 4 April and 15 June. Table 4 summarizes characteristics of these clusters. The number of clusters with RR > 1 increased to five. More districts (52) were included in these emerging clusters with 14 districts contained by high-risk clusters (RR ≥ 2.13) and the other 38 districts were considered low-risk clusters. The most likely high-risk cluster—i.e., **Cluster 1**—with the highest RR of 20.07, contains, collectively, the Dhaka metropolitan area and suburban Dhaka. The second relatively higher risk cluster (**Cluster 13**) contained the neighboring Faridpur (RR = 4.54) and Rajbari district (RR = 1.22). The third relatively higher risk cluster (**Cluster 3**) with an RR of 2.97 contained five districts that are located near Dhaka: Narayanganj (RR = 3.26), Munshiganj (RR = 6.42), Madaripur (RR = 2.4), Shariatpur (1.24), and Chandpur (RR = 1.14). The fourth higher-risk cluster (**Cluster 2**) with an RR of 2.72 included three south-eastern districts: Chittagong (RR = 2.66), Cox’s Bazar (RR = 2.94), and Bandarban (RR = 0.91). The fifth higher-risk cluster (**Cluster 6**) with an RR of 2.13 contained Comilla (RR = 2.06), Noakhali (RR = 2.05), and Feni (RR = 2.3). Figure 6 shows that thirty-nine northwestern, northeastern, southern, and southwestern districts belonged to relatively lower-risk clusters (RR <= 3.0). There were twelve non-emerging COVID-19 risk districts in Bangladesh at the time of this analysis, including Bogra, Gazipur, Gopalganj, Joypurhat, Khagrachhari, Kishoreganj, Kushtia, Manikganj, Netrakona, Pabna, Rangamati, and Sunamganj.

Table 4 shows that, emerging space-time clusters manifest varying onset time and duration. Among the high-risk clusters, Cluster 1, which contains Dhaka megacity and the suburban area, emerged as the major hotspot on May 13 and remained so at the time of this analysis. **Cluster 11** was the longest (6 May–15 June) active cluster over the period of this analysis (4 April–15 June). This cluster contained two relatively lower-risk COVID-19 districts in the north-eastern Bangladesh: Sylhet and Maulvibazar. **Cluster 4**, **5**, **7**, **8**, **9**, **and 12** are the second-longest clusters with lower RR districts. Cluster 1 and 2 were the third-longest (i.e., 34 days or around 5 weeks) active high-risk cluster that are primarily characterized by industrial (e.g., manufacturing industries, seaports) and tourist areas (e.g., sea beaches, hill resorts). Cluster 13 was the most recently formed high-risk cluster that included two neighboring districts: Faridpur and Rajbari. **Cluster 10** was also a recently formed clusters with relatively lower-risk districts in southern Bangladesh that included areas of the Sundarbans mangrove forest.

### 3.3. Progression of Relative Risk of COVID-19 in Bangladesh

#### 3.3.1. Comparison between Early and Later Phase (Pre- and Post-30 April)

**Figure 7:**
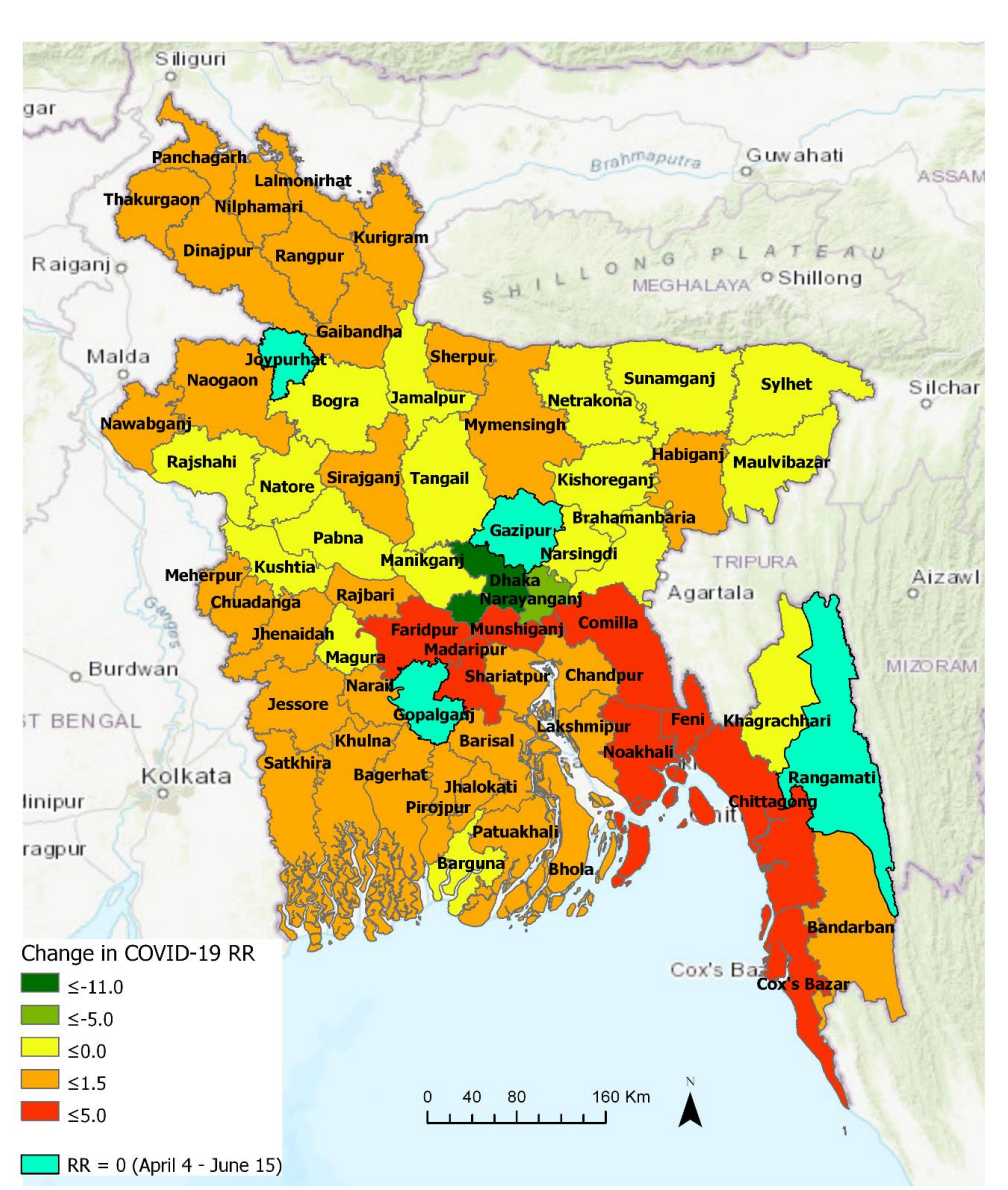
Changes in relative risk of COVID-19 between two emerging periods (4 April–30 April and 4 April–15 June) in Bangladesh.

**Figure 8:**
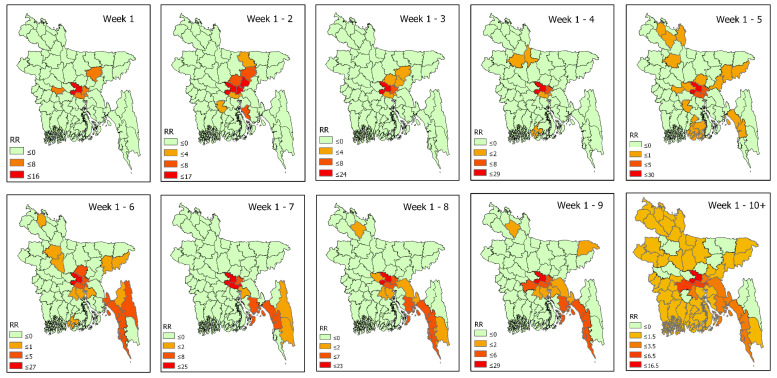
Weekly pattern of space-time propagation of COVID-19 relative risk in Bangladesh.

Figure 7 highlights the changes in the relative risk (RR) of COVID-19 in Bangladesh at the district level between two emerging periods: 4 April–30 April and a longer period of 4 April–15 June. In essence, this temporal change in RR indicates space-time patterns of transmission, most likely responding to spatiotemporally variable levels of non-pharmaceutical intervention measures practiced by people. Out of the sixty-four districts in Bangladesh, only four districts manifested RR = 0 over the two periods. Thus, they were categorized as “non-emerging” COVID-19 regions, although it should be noted that all of these four districts (Gazipur, Gopalganj, Joypurhat, and Rangamati) have experienced COVID-19 outbreak (i.e., > 75 cases in each of these districts) and some of them became emerging clusters (with elevated RR) at some point in time when scanned over a shorter temporal window (see Figure 8). Besides, different parameterization for the space-time scanning, including the duration of emerging clusters and the minimum number of confirmed cases, may provide slightly different patterns.

The suburban region of Dhaka, which is characterized as a high-density working-class population, emerged as a new high-risk cluster with an RR of 16.0 during 1 May–15 June, likely responding to a large-scale violation of social distancing in late April. Although Dhaka metropolitan area experienced a reduced level of risk (i.e., -11.9 change in RR), it remained the epicenter of outbreak with the highest risk level (RR = 16.54) over the same period. Similarly, the relative risk in Narayanganj and Kishoreganj districts decreased as well, although Narayanganj with RR = 3.26 remained one of the high-risk districts. The areas that manifested >3.00 increase in RR over this period were: Faridpur and Munshiganj. Districts with >2.00 increase in RR were: Madaripur, Comilla, Feni, Chittagong, and Cox’s Bazar, followed by Chandpur, Shariatpur, and Noakhali, where RR increased by 1. The relative risk of COVID-19 increased all over the country after 30 April, as seen in Figure 7. This essentially portrays a local community-level transmission all around Bangladesh. It is noticeable that the relatively higher risk areas were spread all around with the highest risk detected in the central, south-central and south-eastern districts. Whereas, some north-eastern, north-western, and southern districts progressed toward reduced risk of an outbreak. Hilly district Khagrachari turned into “non-emerging” areas with RR dropped to 0, whereas another hilly district Bandarban experienced elevated risk with +60 cases. This relatively lower to no risk pattern in the hilly districts could be related to lower population density and restricted movement patterns due to inaccessible locations characterized by high altitude than majority of Bangladesh lands.

#### 3.3.2. Space-Time Propagation of COVID-19 Transmission in Bangladesh

Figure 8 shows space-time progression of COVID-19 disease in Bangladesh at weekly intervals. Based on the available data, ten weekly intervals were used for space-time scanning between 4 April and 15 June. Most of the districts had RR = 0 in week 1, except for Dhaka (mostly metropolitan area) and its industrial belts in Narayanganj, Munshiganj, Kishoreganj, and Rajbari. It should be noted that many Bangladeshi expatriates from Europe and the Middle East returned in Dhaka and subsequently returned to their home districts by early April. Besides Dhaka and its contiguous neighbors being the epicenter of the COVID-19 outbreak, the emergence of Kishoreganj and Rajbari as high-risk areas at this relatively early-phase could be related to the presence of returnees from foreign countries and the epicenter itself. Week 2 shows expansion as well as an increased level of risk toward the north and east of Dhaka to include Gazipur and Narsingdi. Additionally, Netrakona, Gopalganj, and Lakshmipur emerged as high-risk areas. Week 3 is characterized by lower-risk areas, which may well just reflect the missing data issue. Later weeks show how COVID-19 transmitted to more areas with elevated risk. By week 5 (2–8 May), COVID-19 hotspots reached northeastern Bangladesh. In the following weeks (9 May–5 June), southern coastal and hilly districts in southeastern Bangladesh emerged as hot-spots. Although the Bangladesh government lifted nationwide lockdown on May 31 for economic reasons, Figure 8 clearly shows the existence of active hotspots, particularly in the industrial belts of Dhaka and Chittagong by the end of week 9 (i.e., up to 5 June). However, the following week manifested a widespread emergence of COVID-19 infections and elevated outbreak risk in Bangladesh. Figure A4 further highlights an increasing number of districts in Bangladesh experiencing elevated level (i.e., RR > 1) of COVID-19 outbreak risk by 15 June.

With an understating of the weekly progression of emerging clusters, we can begin to link these emergences with intervention scenarios in accordance with the incubation period, which can be up to 2 weeks. Table 5 shows some currently available empirical evidence that may highlight a potential link between detected space-time risk progression in Figure 8 and public events associated with social-distancing measures. An average incubation period of 2–3 weeks between the exposure and emergence events might support these linkages. However, the emergence of Rajbari as a high RR district on the week of 4–10 April may not be directly linked to the shutdown of Daulatdia brothel. In contrast, coronavirus in Rajbari could have been transmitted by Bangladeshi workers returning from Italy or elsewhere. On the other hand, temporal duration between the emergence of COVID-19 risk in the south-eastern coastal districts and the prior tourist activity violating intervention measure in late March is longer than the usual incubation period of two weeks. However, this somewhat ‘delayed’ emergence may have resulted from a low testing rate of potential patients as well as the presence of asymptotic cases in Bangladesh. As more spatial epidemiological evidence and more local-level (e.g., sub-district) data become available, a large-scale contact tracing of confirmed cases will likely confirm these and find additional linkages on the local and inter-regional scales.

## 4. Discussion

This study leveraged prospective space-time scan statistics for identifying currently active or emerging clusters of COVID-19 in Bangladesh. We conducted this analysis at the district level for Bangladesh and the thana level for the Dhaka metropolitan area—one of the most densely populated megacities in the world. This study, to the best of our knowledge, is a first attempt to showcase the space-time progression of COVID-19 risk in Bangladesh. It is also the first work that utilized prospective scanning statistics to detect emerging clusters of COVID-19 in the country. As the COVID-19 cases were still on the rise at the time (mid-June) of this study, with more real-time data becoming available, the prospective approach demonstrated here can be utilized for rapidly monitoring evolving space-time patterns of COVID-19 (or any infectious disease in general) risk in Bangladesh and elsewhere. This will enable government and health officials to take appropriate and time-sensitive intervention measures by considering disease’s space-time diffusion pathways and potentially prepare for future outbreaks of a highly contagious disease.

We investigated emerging space-time cluster patterns of COVID-19 at the thana level in Dhaka. Utilizing available 7 April–15 June, 2020 daily COVID-19 reported cases, we have identified 11 emerging clusters with 23 relatively higher magnitudes of risky thanas (Figure 4). Multiple emerging clusters were spreading all over the city with varying size and magnitude. Most densely populated and lower-to-medium-income locations of Dhaka city around Mirpur, Uttara, Mugda, Rampura, Mohammadpur, Ramna, Shahjahanpur, Gendaria, and Shyampur thana were experiencing a moderate to higher level of risk till mid-June). Among higher-income areas, Dhanmondi and Banani were exhibiting a higher risk of COVID-19 outbreak. However, many thanas were at low to no risk by 15 June, such as Kamrangir Char, Bangshal, Gulshan, which were higher risk areas over the early April to mid-May period. As time progresses and more data become available, using this space-time scanning measure at relatively shorter temporal intervals would assist monitoring the day-to-day or week-to-week transmission of previously existing and newly emerging clusters. Further evaluation with covariates such as age, sex, income, mitigation measures could enable a better understanding of the rate and process of COVID-19 transmission. However, such an analysis was beyond the scope of this study due to data scarcity.

While exploring the space-time progression of COVID-19 cases at the district level, we found that there were only two high-risk clusters in four districts in the central Bangladesh before May. This was most likely introduced by travelers coming from abroad, including China, Italy, the UK, Saudi Arabia, Kuwait, Bahrain, and India. However, although that is the most likely case, as suggested by the chronicle of government press releases and newspaper reports, without rigorous contact tracing of COVID-19 patients in the early phase (e.g., January-February), any such assumption could not be scientifically validated. The Bangladesh government-initiated measures on safe evacuations of Bangladeshi citizens from Wuhan, China, travel restrictions, and social distancing measures may have been effective in restricting further outbreak until early March. However, very insufficient and limited testing facilities may have been contributed to that perceived scenario as well in the country. In addition, large-scale violations of the social distancing effort a few times, particularly between 27 April and 30 April, contributed to the transmission outside of Dhaka. Our analysis identified a substantial increase in the number of emerging clusters in the second phase after 30 April. The onset and duration of the emerging clusters (Table 3 and Table 4) suggest that, Dhaka’s suburb and neighboring regions, where garment workers and other manufacturing industries workers are located, emerged as high-risk clusters exactly within 3 to 4 weeks after a social-distancing breakdown. To better understand the COVID-19 transmission dynamics, datasets on patient’s travel and contact history need to be incorporated, which is very difficult to get at this point in time for a data-sparse country.

**Figure 9:**
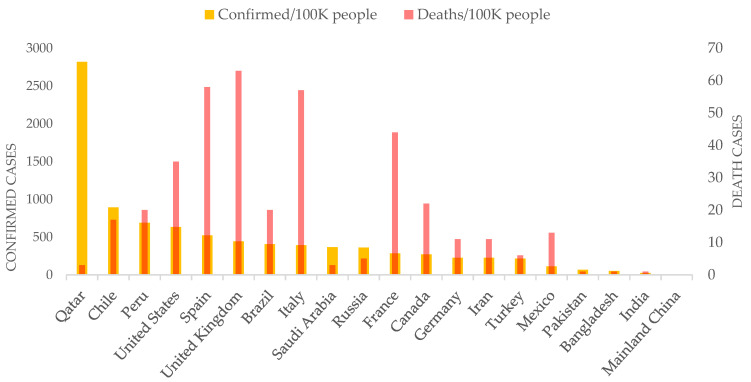
Number of COVID-19 confirmed and death cases per 100,000 people among the top 20 counties with the highest number of confirmed cases (up to 14 June, 2020; Data source: CNN health [41]).

**Figure A3:**
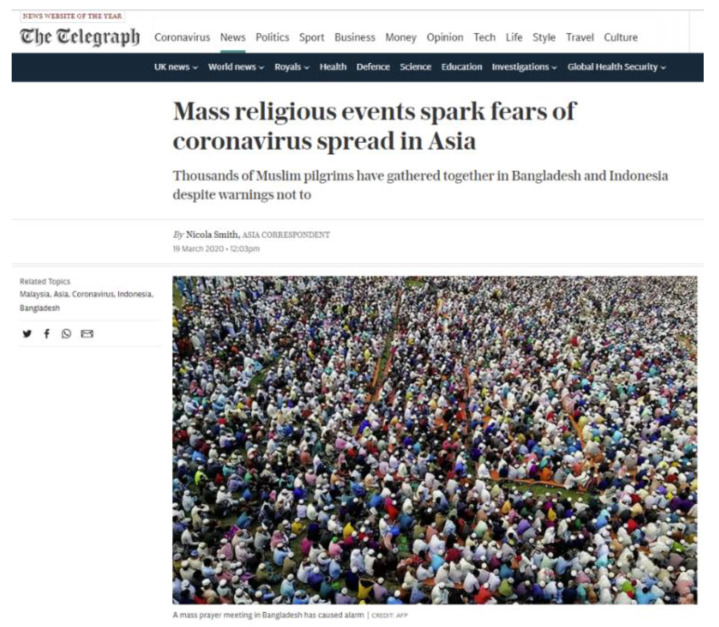
Mass congregation for ritual purpose at the early phase of the COVID-19 outbreak in Bangladesh (18 March).

**Figure A4:**
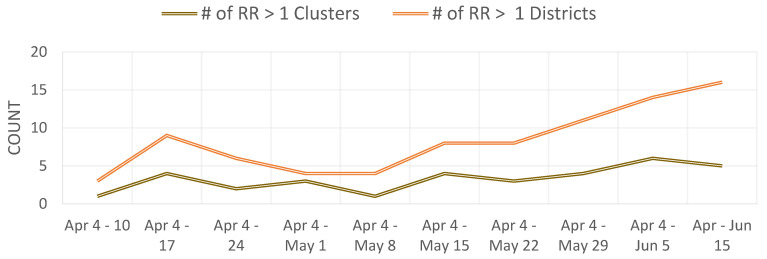
Weekly count of COVID-19 clusters and locations with relative risk greater than 1.

The prospective space-time scanning results presented here suggest that Bangladesh may well have experienced a community-level exposure to the deadly coronavirus pandemic in March. The primary transmission agents were people returning from abroad in February and March, especially from highly infected countries in Europe (e.g., Italy) and the Middle East (e.g., Saudi Arabia). Although the government started taking initiatives in March, it was belated to contain the outbreak primarily in Dhaka city. For that, early detection and effective isolation measures were necessary. In addition, the space-time progression from early April that highlights community-level spread in districts was potentially facilitated by the large-scale violations of social distancing orders. Despite mass gatherings due to poor education as well as behavioral, and dogmatic religious practices (Figure A3), the government’s poor policy guidance and lackluster enforcement exacerbated the COVID-19 situation. Although there are substantial differences in education and socio-economic structure, Bangladesh’s COVID-19 spread pattern could be compared to some of the developed nations such as the United States [5]. Similar to the US cases, the Bangladesh government wasted precious time in the early phase of this pandemic for early detection and isolation, as well as missed the opportunity to rapidly organize a somewhat ‘ineffective’ public health system. Consequently, both countries experienced a rapid outbreak at the city to local levels. However, Bangladesh remains low in coronavirus-death rate per 100,000 people among top-20 countries with the highest counts of confirmed cases by mid-June (Figure 9). The overall lower death rate could be associated with the age composition of the affected people in Bangladesh. By mid-June, around 57% of the confirmed cases in Bangladesh fall into the 21–40 age group, whereas age group 41–60+ constitutes 35% of the cases. At the same time, Bangladesh’s percentage of the younger population aged between 10 and 40 is higher than that of the US, Italy, and France, where most COVID-19 deaths constitute the 40+ age group. However, this observed differences in death rate could not only be explained by demographic differences, but also by potential bias in accounting and reporting COVID-19-related deaths in Bangladesh. Bangladesh is currently facing a deep humanitarian crisis as the broken health care and economic systems are not allowing for the equal treatment of its citizens [39]. Coordinated and targeted NPI measures could help significantly reduce transmission to the local community, as evidenced in the case of China where COVID-19 originated [40].

The timeliness and strength of this study lie in its contribution to rapid surveillance measures as manifested by the prospective space-time scan statistics approach. The prospective version of SaTScan is an excellent exploratory statistic approach for closely monitoring disease outbreaks [42]. However, some limitations can be addressed in future studies when more data become available. First, we only included confirmed cases reported by the IEDCR. The inclusion of suspected and probable cases as additional covariates could improve the understanding of the true magnitude of risk. However, such data were not available or accessible at this point in time. Second, our analyses could not adjust for the age since age-specific data on COVID-19 cases were not available. However, experience worldwide has shown that COVID-19 can affect all age groups, although the recovery rate tends to vary among those groups [5]. It could provide valuable insights into the space-time transmission rate and process through including age-specific data with other medical (pre-existing conditions) and socio-economic indicators (e.g., housing density, median income) at the local level. Third, our findings may suffer from a low COVID-19 testing rate (513 per 1 million; WHO Bangladesh situation report, May 04, 2020) against its high density and the size of the population in Bangladesh [43]. However, as more missing and new data are added, the prospective scan statistic in this research will potentially detect new emerging clusters that would reveal more updated space-time transmission dynamics.

## 5. Conclusions

This study provides novel insights into the COVID-19 transmission dynamics and impacts of NPI scenarios—e.g., social distancing in Bangladesh. We found that the central and south eastern regions in Bangladesh are currently exhibiting a high risk of COVID-19 transmission. Dhaka megacity has remained the highest-risk “active” cluster since early April. The space-time progression of COVID-19 infection was validated against the chronicle of government press releases and newspaper reports, which suggests that Bangladesh has experienced a community-level transmission at the early phase (i.e., March, 2020) primarily introduced by Bangladeshi citizens returning from coronavirus-affected countries in Europe and the Middle East. We also identified potential linkages between the violation of NPIs and the emergence of new higher-risk clusters over the post-incubation periods around Bangladesh.

Overall, the space-time emerging patterns and change in the relative risks of the COVID-19 outbreak identified here can be further compared and validated with local-level epidemiological and travel network datasets. Thus, our study should be taken as a benchmark to understand and unraveling complex pattern and process of COVID-19 transmission in Bangladesh and elsewhere as more heterogeneous data become available and rigorous cross-disciplinary research is conducted in the future. Meanwhile, the knowledge derived in this study on Bangladesh highlights three specific guidelines for reducing disease transmission and minimizing impacts, particularly in the developing countries: (i) in the face of a highly likely disease outbreak, act early to prevent or minimize infection cases by putting necessary travel restrictions, (ii) coordinate with local civic and religious leaders to control human mobility—e.g., cultural or religious gatherings, sporting events, etc.—and (iii) take early preparations on the NPI measures as there are generally inadequate hospital and health care facilities in resource-scarce countries.

## Figures and Tables

**Table 1:** Parameter setup for the prospective Poisson space-time scan statistic.

Parameters Used in Prospective Analysis	
Probability model	Discrete Poisson
Scan for areas	High or low rates
Time aggregation units	1
Spatial window	10% of the population at risk
Spatial window shape	Circular
Maximum temporal cluster size	50% of the study period
Minimum temporal cluster size	2 units
Minimum number of cases for high rate clusters	3 (5 for Dhaka city)
*p*-value	Default
Maximum Monte Carlo replications	999
Spatial and temporal trend adjustments	None

**Table 2:** Emerging space-time clusters of COVID-19 in Dhaka city from 7 April–15 June 2020.

Cluster	Duration (Days)	No. of Thanas	*p*	Observed	Expected	Relative Risk (RR)	No. of Thanas RR > 1
1	30-May–15-June	1	<0.001	1289	107.98	**11.94**	1 (Mirpur)
2	30-May–15-June	6	<0.001	1304	294.99	**4.42**	6
3	30-May–15-June	5	<0.001	1142	332.11	**3.44**	5
4	30-May–15-June	1	<0.001	506	106.85	**4.74**	1 (Uttara)
5	30-May–15-June	2	<0.001	539	163.76	**3.29**	2
6	12-May–15-June	3	<0.001	177	664.8	0.27	0
7	30-May–15-June	5	<0.001	648	300.39	**2.16**	5
8	30-May–15-June	2	<0.001	143	38.5	**3.71**	2
9	12-May–15-June	1	<0.001	152	305.51	0.5	0
10	12-May–15-June	5	<0.001	262	428.88	0.61	1
11	12-May–15-June	1	<0.001	97	203.16	0.48	0

*Note:* RR > 1.0 are highlighted in bold texts.

**Table 3:** Emerging space-time clusters of COVID-19 at the district level between 4 April and 30 April 2020.

Cluster	Duration (Days)	No. of Districts	*p*	Observed	Expected	Relative Risk	No. of Districts RR > 1
1	18-April–30-April	3	<0.001	3866	310.1932	**25.44**	3
2	18-April–30-April	6	<0.001	56	349.8174	0.15	0
3	18-April–30-April	6	<0.001	68	332.197	0.20	0
4	18-April–30-April	6	<0.001	48	288.4021	0.16	0
5	18-April–30-April	4	<0.001	73	267.9812	0.27	0
6	18-April–30-April	8	<0.001	100	281.342	0.35	0
7	18-April–30-April	6	<0.001	126	318.2578	0.39	0
8	18-April–30-April	4	<0.001	45	126.2793	0.35	0
9	18-April–30-April	1	<0.001	147	71.30506	**2.08**	1
10	22-April–30-April	2	<0.001	34	89.81475	0.38	0

Note: RR > 1.0 are highlighted in bold texts.

**Table 4:** Emerging space-time clusters at the district level, between 4 April and 15 June 2020.

Cluster	Duration (Days)	No. of Districts	*p*	Observed	Expected	Relative Risk (RR)	No. of Districts RR > 1
1	13-May–15-June	2	<0.001	18,314	1360.11	**20.07**	2
2	13-May–15-June	3	<0.001	4828	1879.79	**2.73**	2
3	22-May–15-June	5	<0.001	3421	1202.36	**2.97**	5
4	11-May–15-June	5	<0.001	256	1808.05	0.14	0
5	11-May–15-June	7	<0.001	946	2510.26	0.37	0
6	22-May–15-June	3	<0.001	2745	1327.34	**2.13**	3
7	11-May–15-June	3	<0.001	346	1377.94	0.25	0
8	11-May–15-June	2	<0.001	210	1112.93	0.19	0
9	11-May–15-June	7	<0.001	927	2099.74	0.43	0
10	6-Jun–15-June	8	<0.001	984	2018.82	0.48	0
11	6-May–5-June	2	<0.001	2	308.89	0.01	0
12	11-May–15-June	4	<0.001	1112	2032.00	0.54	0
13	7-Jun–15-June	2	<0.001	444	133.02	**3.36**	2

*Note:* RR > 1.0 are highlighted in bold texts.

**Table 5:** Potential linkages between space-time propagation of COVID-19 risk and social-distancing violation in Bangladesh.

Onset Week	High RR District	Intervention Scenarios			News Reference(s)
		*What* Happened	*When* Happened	Likely Incubation Period	
4–10 April	Rajbari	Daulatdia brothel–largest in Bangladesh, was shut down	20 March	~2 weeks	India Today (21 March 2020) [34] Karim, N. (23 March 2020) [35]
11–17 April	Lakshmipur	>25,000 people congregated for a prayer event called ‘Khatme Shifa’ at the Central Eidgah in Raipur	18 March	~3 weeks	AFP News (19 March 2020) [36]
2 May–8 May	Brahmanbaria Habiganj Maulvibazar	>100,000 people attended a funeral in Brahmanbaria	18 April	~2 weeks	Mahmud & Garcia (20 April 2020) [37]
	Chittagong	Coastal areas of Chittagong and Cox’s Bazar bustled with people and activity	Imposed school holiday (18–28 Mar)	~5 weeks	Nooruddin & Shahid (31 March 2020) [38]
9–15 May	Madaripur Shariatpur Chandpur Feni Cox’s Bazar	Amidst nationwide lockdown, people rushed to Dhaka’s industrial belts to resume working	27 April	~ 2 weeks	Nooruddin & Shahid (31 March 2020) [38]
